# Prevalence of Anisakiasis in Madrid (Spain) after 20 Years of Preventive Legislation

**DOI:** 10.3390/pathogens13090782

**Published:** 2024-09-11

**Authors:** Eva Blanco-Costales, Alejandra L. González-Quevedo, Laura Lorenzo-Bernardo, María P. de la Hoz-Martín, Marta Rodero, Pilar Puente, Irene Moreno-Torres, Carmen Cuéllar, Juan González-Fernández

**Affiliations:** 1 Unidad de Parasitología, Departamento de Microbiología y Parasitología, Facultad de Farmacia, Universidad Complutense de Madrid, 28040 Madrid, Spain; evblan03@ucm.es (E.B.-C.); alelop15@ucm.es (A.L.G.-Q.); lloren06@ucm.es (L.L.-B.); mdelahoz@ucm.es (M.P.d.l.H.-M.); mrodero@ucm.es (M.R.); 2 Unidad de Control de Calidad, Centro Militar de Farmacia de la Defensa, Base Logística San Pedro, 28770 Colmenar Viejo, Spain; 3 Unidad de Enfermedades Desmielinizantes, Servicio de Neurología, Hospital Universitario Fundación Jiménez Díaz, 28040 Madrid, Spain; irene.morenot@quironsalud.es

**Keywords:** *Anisakis*, blood donors, Regulation (EC) No. 853/2004, RD 1420/2006, IgG, IgE, IgA, ELISA

## Abstract

Historical seroprevalence data for *Anisakis* in Spain vary greatly depending on the sampling region owing to different fish consumption habits. As a result of European Regulation (EC) No. 853/2004, the Royal Decree 1420/2006 on the prevention of parasitosis by *Anisakis* in fishery products supplied by establishments that serve food to final consumers or to communities came into force in Spain. In this study, a prevalence study of *Anisakis* in Madrid has been conducted to verify the prophylactic effects of the application of the law. Sera from 500 blood donors from the Fundación Jiménez Díaz University Hospital (Madrid/2021–2023) were collected, and the levels of anti-*Anisakis* IgG, IgA, and IgE were analyzed by ELISA, comparing them with those obtained with 110 donors from the Red Cross and the “Gómez Ulla” Central Defense Hospital (Madrid/2001–2002). The percentages of positivity in the 2021–2023 donor group were IgG (13.6%), IgA (13.6%), and IgE (2.2%), while in the 2001–2002 donors they were positive for IgG (15.45%), IgA (14.54%), and IgE (11.65%). A reduction of more than 80% was observed in the prevalence of anti-*Anisakis* IgE in the healthy population of Madrid, which confirmed the positive effect of RD1420/2006, which was later incorporated into RD1021/2022.

## 1. Introduction

The majority of species within the Anisakidae family are distributed across three genera of parasitic nematodes that infect aquatic vertebrates: *Anisakis*, Dujardin, 1845; *Contracaecum*, Railliet and Henry, 1913; and *Pseudoterranova*, Krabbe, 1878. The definitive hosts of these parasites are marine mammals (cetaceans and pinnipeds) and fish-eating birds. The paratenic or intermediate hosts are fish, squids, and other invertebrates, whereas the first intermediate hosts are crustaceans. Some species within these genera have the potential to infect humans accidentally, but the majority of documented cases are associated with *Anisakis* spp. [1,2].

The disease caused by *Anisakis* spp. is known as anisakiasis, and its physiopathological mechanisms depend on the amount of IgE produced and on the local inflammatory reactions caused by larval penetration of the gastrointestinal mucosa when consumed in raw or undercooked marine fish [3]. It can be classified into different clinical entities according to the localization of the larvae: gastric, intestinal, or ectopic/extraintestinal anisakiasis, or according to symptoms: gastroallergic anisakiasis or *Anisakis* sensitization-associated chronic urticaria [4,5,6]. In all cases, when live *Anisakis* larvae penetrate, specific IgE is produced [6,7,8,9]. In a previous study carried out in Madrid with donor sera collected in 2002, the prevalence of anisakiasis by specific IgE was 12.4%. It was related to the consumption of raw fish, especially anchovies in vinegar, which is in contrast to the low prevalence observed in Galicia (0.43%) [10]. Epidemiological studies have demonstrated that the prevalence of anisakiasis, as determined by the presence of specific IgE, is not related to fish consumption per capita because in Madrid, fish consumption is lower than in Galicia (79 g vs. 111 g) [11,12]. Similarly, in Norway, the prevalence is 2%, but the fish consumption per capita is 163 g [13,14]. Neither in Galicia nor in Norway are there raw or undercooked fish-eating habits, and if these habits exist, the fish is frozen before eating.

There has been considerable debate regarding the classification of *Anisakis* as a food allergen or accidental human parasite [8]. However, research has demonstrated that the antigenic proteins of dead larvae (found in cooked or frozen fish) exposed in the gastrointestinal tract are insufficient to degranulate mast cells and trigger allergic symptoms [7,15,16]. Allergic symptoms, if present, are only due to active parasitism and not due to the ingestion of *Anisakis* antigens from dead larvae, as is the case with classical food allergens [8]. Therefore, *Anisakis* is considered a frontier between allergy and parasitism [6].

In 2004, Regulation (EC) No. 853/2004 of the European Parliament and of the Council of 29 April 2004, laying down specific hygiene rules for food of animal origin, came into force in Europe [17]. Thus, Spain, as a member state, established a national law to achieve the objectives of that regulation. This law was the Royal Decree (RD) 1420/2006, which came into force in Spain on 19 December 2006 [18]. It includes the obligation for the owners of establishments serving food to final consumers or collectivities (bars, restaurants, cafeterias, hotels, hospitals, schools, residences, company canteens, catering companies, and similar) to freeze the fish products (including pickled, salted, or cold smoked products in which the central temperature of the product has not exceeded 60 °C) that are going to be consumed raw, at a temperature of −20 °C or lower for at least 24 h. At present, this RD has been repealed to be included in a broader one in which new details appear, such as a minimum of 15 h of freezing at −35 °C, the inclusion of live bivalves, the exclusion of fishery products from inland waters, and marine aquaculture in which fish are not fed with food that may contain live larvae [19].

According to the re-evaluation of the 2010 reports recently made by the European Food Safety Authority (EFSA) using new data on the risk of anisakiasis from consumption of fishery products in Europe, freezing at a core temperature of −15 °C for at least 96 h, −20 °C for at least 24 h, or −35 °C for at least 15 h, and heating above 60 °C for one minute, remain the most efficient methods to kill larvae. Farmed marine fish are less likely to present larvae, but their absence cannot be confirmed; thus, these products must not be consumed raw or undercooked [9]. 

Even though the law exists and is in force, EFSA and the European Centre for Disease Prevention and Control (ECDC) reported in 2020 that two outbreaks in Spain were caused by *Anisakis*. One of these involved three individuals were parasitized due to the failure to freeze raw anchovies pickled in vinegar [20]. In addition, people can buy fresh parasitized fish and prepare it at home to be ingested raw by traditional procedures, with a high risk of infection by live larvae.

In this study, we updated the prevalence of *Anisakis* in Madrid, Spain, obtaining sera from healthy blood donors and comparing them with sera with similar characteristics analyzed 20 years ago to test the prophylactic effect of the law. 

## 2. Materials and Methods

### 2.1. Sample Size Calculation

The sample size was calculated using sample size for frequency in a population, available online through OpenEpi.com [21]. The total population size corresponding to the Hospital Universitario Fundación Jiménez Díaz, Madrid (HUFJD) was estimated to be 800,000 according to the annual report in 2022 (https://www.comunidad.madrid/sites/default/files/doc/sanidad/memo/memoria_2022_hu_fundacion_jimenez_diaz_ok.pdf, accessed on 1 September 2024). An estimated prevalence of 12.4% was considered based on a previous study [10]. The minimum sample size calculated was 167 random blood donors for a 95% confidence interval.

### 2.2. Sera from Healthy Blood Donors and Questionnaire 

Blood was collected from 500 donors at the Blood Bank of the Hospital Universitario Fundación Jiménez Díaz, Madrid (HUFJD), Spain, as part of the clinical study PIC142-21_FJD approved by the Research Ethics Committee of the same hospital. A single sample collection was performed from 2021 to 2023 using the same route used for voluntary blood donation at the hospital.

Donors were informed about the objectives and doubts they had answered before agreeing to participate in the study. The participants signed an informed consent form, and afterward we requested the following information: date of birth, sex, “How many times do you eat fish during the week?”, and “How many times have you consumed raw fish during the year?”.

Sterile 8.5 mL tubes (PET BD Vacutainer^®^, Becton, Dickinson and Company, Franklin Lakes, NJ, USA) with the blood obtained from each subject were centrifuged for 10 min at 1500× *g*. After centrifugation, the serum was transferred to freezing tubes, labeled, and codified. Finally, they were stored at −80 °C. 

Previous data from 110 sera from healthy blood donors of the Red Cross and the Hospital Central de la Defensa “Gómez Ulla” (RC-HCD), Madrid, Spain, collected from 2001 to 2002, were used in the study to analyze the evolution of the prevalence of *Anisakis*. 

### 2.3. Anisakis Crude Extract Preparation

*Anisakis simplex* sensu lato third-stage larvae (L3) were picked up manually from the viscera, flesh, and body cavities of naturally infected blue whiting (*Micromesistius poutassou*) from fishing grounds in the Eastern Atlantic, specifically off the Portuguese continental shelf, and the Western Mediterranean basin. L3 were exhaustively washed in water to carry out the morphological identification. L3 crude extract was prepared according to a standard procedure [22]. Antigens were extracted by manual mechanical homogenization in a mortar from L3, which were manually selected from fresh *Micromesistius poutassou*. PBS (phosphate-buffered saline) was added until a homogeneous mixture was obtained. Subsequently, the extract was sonicated six times on ice for 10 s. For protein extraction, it was kept with PBS in the refrigerator at 4 °C overnight. The next day, the extract was centrifuged at 6700× *g* for 1 min to separate the tissue debris. The supernatants were processed by removing lipids with *n*-hexane at a ratio of 3 mL hexane/7 mL extract. Another centrifugation step was performed at 6700× *g* for 1 min to accelerate phase separation, and the aqueous phase was collected. 

Finally, on 12–14 kDa membranes (Medicell Intl. Ltd., London, UK), dialysis was performed against PBS at 4 °C to subsequently titrate the protein content using the Bradford method (Bio-Rad Laboratories GmbH, Munich, Germany).

### 2.4. Anti-Anisakis IgG, IgA, and IgE Determination by ELISA

Enzyme-Linked ImmunoSorbent Assay (ELISA) was used to measure serum antibodies in 96-well plates (Costar™ 96-Well, Corning, NY, USA). 

*Anisakis* antigen was diluted at 10 μg/mL in 0.1 M carbonate/bicarbonate buffer (pH 9.6). Subsequently, 100 μL/well were incubated overnight at 4 °C for antigen binding to the plate. After each incubation, three vigorous washes with PBS-Tween (0.05% Tween 20 in PBS) were performed.

Second, the plate was blocked with 5% bovine serum albumin (BSA) in PBS, 200 μL/well were added, and the plate was incubated for 1 h at 37 °C. After washing, we diluted the sera in PTB (5% BSA in PBS-Tween). For the determination of IgG and IgA, a 1/100 dilution was used, whereas for the determination of IgE, the necessary dilution was 1/2. Diluted sera were added (100 µL/well) in duplicate and incubated for 2 h at 37 °C. After washing, goat anti-human IgG horseradish peroxidase conjugated (HRP) (Biosource, Camarillo, CA, USA) at 1/8000 dilution in PTB and goat anti-human IgA-HRP (Biosource, Camarillo, CA, USA) at 1/3000 dilution in PTB were incubated for 1 h at 37 °C. The serum IgE levels were determined in two steps: in the first step, an unlabeled mouse anti-human IgE monoclonal antibody diluted at 1/1000 in PTB (IgG1κ, E2A11, INGENASA, Madrid, Spain) was incubated for 2 h at 37 °C, followed by incubation with HRP-conjugated goat anti-mouse IgG1 (Thermo Fisher Scientific, Eugene, OR, USA) at 1/1000 in PTB. 

After washing, 100 µL/well of a 0.04% solution of o-phenylenediamine dichlorohydrate (OPD) (Sigma-Aldrich, St. Louis, MO, USA) in citrate-phosphate buffer (pH = 5) and 0.04% (*v*/*v*) hydrogen peroxide were added. The plate was then placed in the dark for 20 min. Finally, the reaction was stopped by adding 50 µL/well 3 N H_2_SO_4_.

Optical density (OD) was measured at 490 nm using a spectrophotometer (BioTek ELx808™, Winooski, VT, USA). The final values were calculated by subtracting the OD values of the same serum sample in the absence of the antigen. All determinations were performed in duplicate.

For data analyses, serum was considered positive if its OD was higher than X + SD, where X is the mean and SD is the standard deviation of the corresponding group of sera (2001–2002; N = 110 or 2021–23; N = 500) for the same class of immunoglobulin analyzed. 

### 2.5. Statistical Analysis

Pearson’s chi-squared test was carried out to analyze the association of *Anisakis* positivity for each class of immunoglobulin with the sera collection date. Data distribution was evaluated with a Kolmogorov–Smirnov’s test. The Mann–Whitney *U* test was used to compare medians between groups. Spearman’s correlation test was used to assess the relationship between anti-*Anisakis* immunoglobulin levels and fish consumption. All statistical analyses were performed using IBM SPSS Statistics version 29 (IBM Corp., Armonk, NY, USA). Statistical significance was established at *p* < 0.05, 2-tailed. 

## 3. Results

### 3.1. Healthy Blood Donor Groups

Five hundred healthy blood donors from HUFJD participated in this study. Their mean age was 39.6 ± 13.69 years (18–65 years). Of these, 226 (45.2%) were male and 274 (54.8%) were female. Previous results from 110 anonymous sera from RC-HCD with a weighted mean age of 42.38 years (25–60 years) were obtained from our database.

### 3.2. Fish Consumption Habits

**Figure 1:**
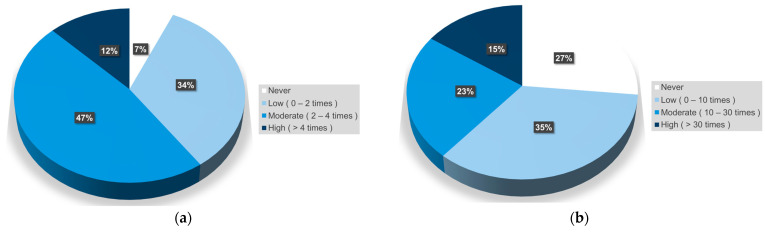
Fish consumption habits of healthy blood donors from the Hospital Universitario Fundación Jiménez Díaz, Madrid (2021–2023, N = 500): (**a**) fish consumption per week; (**b**) raw fish consumption per year.

Based on the survey carried out of the 500 donors from HUFJD while their blood was being collected, it was found that 467 out of 500 (93.4%) consumed fish weekly, and 367 out of 500 (73.4%) ate raw fish throughout the year. The frequency of fish consumption in the study population was classified as low (0–2 times per week or 0–10 times per year), moderate (2–4 times per week or 10–30 times per year), or high (>4 times per week or >30 times per year) based on the number of times the fish were consumed per week and the intake of raw fish throughout the year (Figure 1).

The mean [%95 confidence interval (CI)] frequency of fish consumption was 2.23 [2.07–2.38] times per week, with a median of two times per week. The mean [%95 CI] frequency of raw fish consumption was 16.89 [11.98–21.80] times per year, with a median of 5 times per year.

### 3.3. Anisakis Prevalence

**Figure 2:**
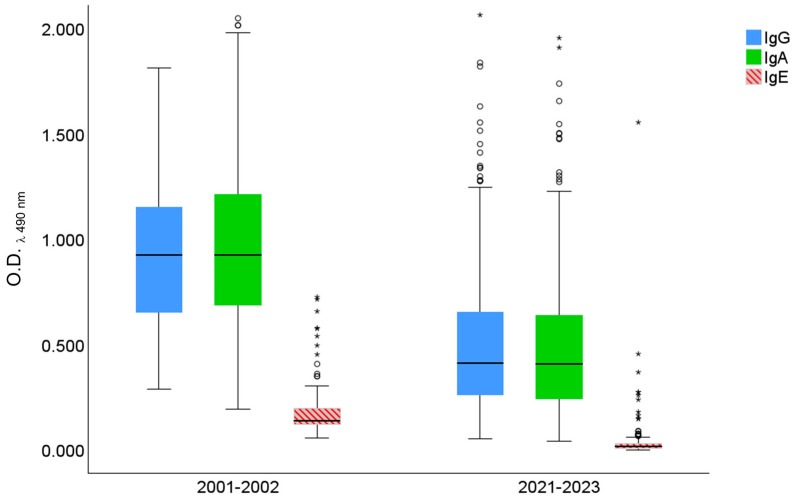
Antibody levels against *Anisakis* antigens in healthy blood donors from the Red Cross and the Hospital Central de la Defensa “Gómez Ulla”, Madrid (2001–2002, N = 110) vs. healthy blood donors from the Hospital Universitario Fundación Jiménez Díaz, Madrid (2021–2023, N = 500). O.D.: optical density at λ = 490 nm. ○ and * indicate outliers.

The 2001–2002 group showed the following mean [%95 CI] OD levels of IgG (0.951 [0.882–1.020]), IgA (1.019 [0.921–1.117]), and IgE (0.190 [0.163–0.216]). Whereas mean [95%CI] OD in the 2021–2023 group decreased for IgG (0.496 [0.467–0.526]), IgA (0.489 [0.460–0.517]), and IgE (0.026 [0.019–0.033]). In the 2001–2002 and 2021–2023 groups, specific IgG and IgA antibody levels were higher than IgE and showed similar patterns (Figure 2).

**Figure 3:**
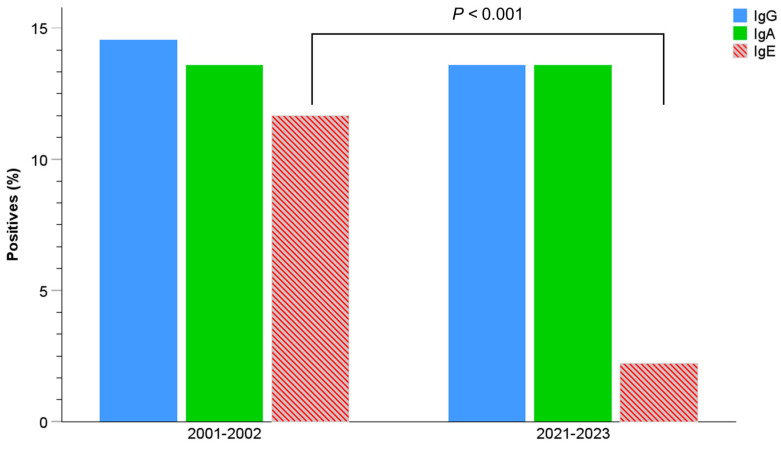
Percentage of positive sera (OD > M + SD) for anti-*Anisakis* IgG, IgA, and IgE in healthy blood donors from the Red Cross and the Hospital Central de la Defensa “Gómez Ulla”, Madrid (2001–2002, N = 110) vs. healthy blood donors from the Hospital Universitario Fundación Jiménez Díaz, Madrid (2021–2023, N = 500) (Pearson’s chi-square test).

After calculating the positivity threshold for each immunoglobulin, we calculated the percentage of positivity. The percentages of IgG- and IgA-positive sera, although exhibiting a slight decline, remained relatively stable in the current donor population when compared to the donors 20 years ago. Conversely, IgE positivity significantly decreased from 11.65% in 2001–2002 to 2.2% in 2021–2023 (*p* < 0.001) (Table 1 and Figure 3). As IgE is the antibody class used to ascertain the prevalence of *Anisakis*, the analyzed population displayed an *Anisakis* prevalence of 2.2%.

The data presented a non-normal distribution (*p* < 0.001); therefore, non-parametric statistical tests were used. In donors from both groups, a positive correlation was observed between specific IgG and IgA levels (*p* < 0.001). Anti-*Anisakis* IgG also correlated positively with IgE (*p <* 0.01). Anti-*Anisakis* IgA levels correlated positively with IgE levels in the group of 2001–2002 donors (*p* = 0.003). 

Anti-*Anisakis* IgG levels correlated positively with the weekly fish intake in the 2021–2023 donor group (*p* = 0.047). All correlations are included in a Supplementary File.

Grouping the donors into positive and negative groups, we observed the IgG-positive group exhibited significantly higher levels of specific IgA (*p* = 0.004 for 2001–2002 donors; *p* = 0.002 for 2021–2023 donors) and IgE (*p* < 0.001 for 2001–2002 donors; *p* = 0.002 for 2021–2023 donors) compared to the anti-*Anisakis* IgG-negative group. Additionally, the IgG-positive group consumed a greater quantity of fish per week (*p* = 0.022) than the IgG-negative group (see the raw data in the Supplementary File).

As expected, individuals with positive levels of anti-*Anisakis* IgA showed higher levels of specific IgG (*p* < 0.002 for 2001–2002 donors; *p* < 0.001 for 2021–2023 donors). 

Lastly, anti-*Anisakis* IgE-positive donors showed higher levels of serum anti-*Anisakis* IgG (*p* < 0.001 for both donor groups) and consumed less raw fish per year (*p* = 0.026). 

## 4. Discussion

This cross-sectional seroprevalence study conducted on samples from 500 blood donors represents the first investigation about anisakiasis in Spain following the implementation of preventive legislation for anisakiasis in 2004–2006.

In Spain, previous reliable data on the prevalence of *Anisakis* were 12.4% in Madrid [10] and 0.43% in Galicia [23]. 

In our study of anisakiasis prevalence (IgE), we found 11.65% in sera from 2001, which is consistent with other prevalence studies [10] (12.4%). Our current data on the anisakiasis prevalence was 2.2% in Madrid, indicating a marked reduction. The anisakiasis prevalence in other European countries was in line with our results: 3–9% in Portugal, 2% in Norway, 0.4–12.7% in Italy, and 2–2.5% in Croatia. The prevalence rates in other non-European countries were also similar to those obtained in our study: 5.1% in Morocco, 10% in Japan, and 5–6.65% in South Korea [12]. Other prevalence studies, which used less specific techniques, such as the Skin Prick Test or CAP-FEIA, or were carried out in different populations, such as patients or fish sector workers, were also carefully reviewed [24].

Many other factors, apart from legislation, mainly related to health education on heating or freezing fish, may have influenced the observed decrease in cases. The current Spanish and European legislative frameworks, in conjunction with the dissemination of news reports alerting the public to the potential presence of *Anisakis* larvae in fish, have sought to enhance awareness of the parasite in Spain. It is a legal requirement for restaurants and catering companies to freeze fish to kill larvae whenever fish is consumed raw [9,18,19]. Therefore, the most probable potential source of infection is consumers of raw fish who prepare marinated or pickled fish at home and do not freeze it beforehand. Given that legal requirements mandate restaurants and catering companies to freeze fish prior to serving in order to inactivate *Anisakis* L3 larvae, the observed reduction in prevalence from approximately 12% to 2% suggests that the Spanish population has adopted effective preventive habits against *Anisakis* infection. 

In addition, it is pertinent to discuss the potential effects of ingesting antigens from dead *Anisakis* larvae present in the cooked/frozen fish we consume daily. Three clinical studies showed that none of the *Anisakis*-positive patients suffered allergic reactions when orally challenged with dead *Anisakis* larvae [7,15,16]. The evolution of anti-*Anisakis* antibody concentrations after *Anisakis* antigens oral intake remains unknown; however, control subjects without urticaria showed detectable levels of anti-*Anisakis* serum IgA (logically less than sensitized patients with acute or chronic urticarial reaction by *Anisakis*) and correlated positively with pro-inflammatory cytokines [25]. In the present study, we also detected IgG and IgA seroprevalences of 13.6%, which are similar to those obtained with the sera of donors obtained 20 years before (15.55% and 14.54%, respectively). This indicates that people continue to be in contact with *Anisakis* larvae antigens, and only ingestion of the larval antigens is enough to develop an immune response with antibody production. In other words, a local digestive immunological response to contact with the allergens is sufficient to maintain specific serum IgG and IgA levels against a parasite that had never parasitized the host (without IgE production). 

Despite the significant findings of this study, several limitations should be considered. This study had a single-center design because financial constraints precluded a multicenter or international approach. However, Madrid was chosen as the optimal study site due to its cosmopolitan nature, with a population of residents from diverse regions of Spain and 19% from other countries. Romania, Colombia, China, and Morocco are the primary countries of origin for immigrants residing in Madrid, each representing a major continental source of migration [26]. This demographic diversity enhances the generalizability of the findings despite geographically limited sampling.

Furthermore, there was a reduced number of sera from the 2001–2002 group, but with a sample size of 110, we reached a 90% confidence level. 

In addition, we did not have clinical data on other comorbidities in the donors due to ethical restrictions, which could influence the observed immunoglobulin levels. However, a previous study [10] was carried out under the same conditions.

Notwithstanding these limitations, our findings indicate that other investigations overestimated the prevalence of anisakiasis in Spain by up to 14.7–18.4%. This was a consequence of the erroneous assumption that a correlation exists between fish consumption and parasitized fish rates and anisakiasis prevalence [27,28]. However, the factors affecting parasitization are not only the exposure level (per capita fish consumption and fish parasitization rates) but, more importantly, fish-eating habits (raw or undercooked fish with live larvae) [11]. 

Regarding fish consumption rate, measured in kg/inhabitant/year, Portugal reported the highest rate in Europe (59.91), followed by Norway (54.56), Japan (46.74), and Spain (46.02) in 2019 [29] (https://oceans-and-fisheries.ec.europa.eu/, accessed on 1 September 2024). The results of our study regarding fish consumption among 500 blood donors indicated that the frequency was approximately twice per week (raw or cooked, fish in general), with a median of five times per year for raw fish. This aligns with our position as the fourth-ranked country in terms of fish consumption. A raw fish consumption survey conducted in Portugal showed that 18/763 (2.36%) did not eat fish, and 421/745 (56.5%) did not eat raw fish [30]. In our population, 7% did not eat fish, and 27% did not eat it raw. Although almost 20% more subjects in our study ate raw fish than those in the Portuguese study, the anisakiasis prevalence was low because the intake was probably frozen fish. In our population, the raw fish intake rate was low at 35% (0–10 times per year), but 81% of our participants had a moderate (1–4 times per week) cooked fish intake. 

Due to the high fish consumption rates and previous prevalence studies, Spain has been postulated to have one of the highest incidences of anisakiasis using models and questionnaires for risk assessment. It is frequently claimed that the number of anisakiasis cases is underdiagnosed and misdiagnosed because of non-specific symptoms and insufficient anamnesis regarding the ingestion of raw or undercooked fish. The relatively low rate of hospitalization among anisakiasis cases, estimated at 1–2% [3], coupled with the prevalence of 2.2% observed in our results, suggests that preventive measures, including legislation and domestic practices such as freezing and cooking fish, are effective in reducing the incidence of infection. A retrospective study of 2471 cases of anisakidosis-related hospitalization in Spain from 1997 to 2015 showed that Madrid had the highest hospitalization rate (9.17 hospitalizations/10^6^ population) [3]. In other European countries affected by the equivalent European Regulation, Italy reported 73 cases between 1996 and 2017 [31], while France documented 37 cases between 2010 and 2014 [32].

In conclusion, with our current results of 2.2% prevalence of anisakiasis in Madrid (Spain) compared with the 11.65% observed before the enactment of the preventive legislation against anisakiasis, we demonstrate the success of prevention programs and compliance with the law. The conserved specific IgG and IgA positivity rates without IgE open new horizons of research on mucosal immunity because dietary *Anisakis* antigens found in fish may elicit a local immune response. 

## Figures and Tables

**Table 1:** *Anisakis* seroprevalence in healthy blood donor sera collected with 20 years of difference (2001/2002–2021/2023).

Population		IgG	Χ^2^	*p*	IgA	Χ^2^	*p*	IgE	Χ^2^	*p*
2001/2002 N: 110	Positives (%; 95% CI)	17 (15.45; 8.59–22.32)	0.259	0.611	16 (14.55; 7.85–21.24)	0.068	0.794	12 * (11.65; 5.35–17.95)	20.791	<0.001
2021/2023 N: 500	Positives (%; 95% CI)	68 (13.6; 10.59–16.61)			68 (13.6; 10.59–16.61)			11 (2.2; 0.91–3.49)		

N: number of cases; CI: confidence interval; Χ^2^: Pearson’s chi-square; *p*: asymptotic significance (2-sided). * N: 103.

## Data Availability

The data presented in this study are available in a Supplementary File and were pseudonymized by the corresponding author J.G.F. due to ethical and privacy reasons.

## Supplementary Materials

The following supporting information can be downloaded at: https://www.mdpi.com/article/10.3390/pathogens13090782/s1. Supplementary File: Pseudonymized data and correlations.
