# Supplementary material for: Prevalence of Anisakiasis in Madrid (Spain) after 20 Years of Preventive Legislation
Source: Pathogens. 2024 Sep 11;13(9):782. doi: 10.3390/pathogens13090782 (PMC11435309; doi:10.3390/pathogens13090782)
Supplement: Supplementary file 1 [file pathogens-13-00782-s001.zip › Correlations (supplemmentary).pdf]

Correlations

Group				IgG	IgA	IgE	Fish week	Raw fish per year
2001-2002	Spearman's rho	IgG	Correlation Coefficient	1.000	0.432**	0.317**		
			Sig. (2-tailed)	.	<0.001	0.001		
			N	110	110	103		
		IgA	Correlation Coefficient	0.432**	1.000	0.288**		
			Sig. (2-tailed)	<0.001	.	0.003		
			N	110	110	103		
		IgE	Correlation Coefficient	0.317**	0.288**	1.000		
			Sig. (2-tailed)	0.001	0.003	.		
			N	103	103	103		
2021-2023	Spearman's rho	IgG	Correlation Coefficient	1.000	0.236**	0.118**	0.089*	0.003
			Sig. (2-tailed)	.	<0.001	0.008	0.047	0.939
			N	500	500	500	500	500
		IgA	Correlation Coefficient	0.236**	1.000	-0.025	0.053	0.001
			Sig. (2-tailed)	<0.001	.	0.572	0.237	0.981
			N	500	500	500	500	500
		IgE	Correlation Coefficient	0.118**	-0.025	1.000	0.029	-0.067
			Sig. (2-tailed)	0.008	0.572	.	0.516	0.135
			N	500	500	500	500	500
		Fish per week	Correlation Coefficient	0.089*	0.053	0.029	1.000	0.294**
			Sig. (2-tailed)	0.047	0.237	0.516	.	<0.001
			N	500	500	500	500	500
		Raw fish per year	Correlation Coefficient	0.003	0.001	-0.067	0.294**	1.000
			Sig. (2-tailed)	0.939	0.981	0.135	<0.001	.
			N	500	500	500	500	500

*. Correlation is significant at the 0.05 level (2-tailed).

**. Correlation is significant at the 0.01 level (2-tailed).
